# Fumigant Toxicity of Essential Oils against *Frankliniella occidentalis* and *F*. *insularis* (Thysanoptera: Thripidae) as Affected by Polymer Release and Adjuvants

**DOI:** 10.3390/insects13060493

**Published:** 2022-05-24

**Authors:** Karim Gharbi, Jia-Wei Tay

**Affiliations:** Urban Entomology Laboratory, Department of Plant and Environmental Protection Sciences, University of Hawaii at Manoa, 3050 Maile Way, Gilmore Hall 310, Honolulu, HI 96822, USA; kgharbi@hawaii.edu

**Keywords:** essential oils, fumigant toxicity, *F. occidentalis*, phytotoxicity, *S. lycopersicum*, polymer release, hydrogel, thrips

## Abstract

**Simple Summary:**

*Frankliniella occidentalis* is among the most economically significant pests of greenhouse crops. In contrast, *F. insularis* is a relatively minor pest with a narrower distribution and host range. We conducted a series of fumigation assays to assess the vulnerability of both species to fumigation with essential oils released from hydrogels. These hydrogels contained either (*R*)-linalool, (*S*)-linalool, racemic linalool, or a binary mixture of (*R*)-linalool with one of twelve other essential oils. *Solanum lycopersicum* seedlings were screened for their sensitivity to the most potent fumigants, as determined from thrips bioassays. The least saturated hydrogels conditioned in essential oils were the most effective, and both species of thrips were more sensitive to (*R*)-linalool than to (*S*)-linalool. *Frankliniella occidentalis* was significantly more resistant to all treatments than *F. insularis*. Treatment of *S. lycopersicum* with the same concentrations of oils required to control thrips resulted in reduced root and hypocotyl lengths, most severely in seedlings exposed via foliar sprays than as fumigants. While our study demonstrates that essential oils are a promising alternative to conventional insecticides for thrips control, the resistance demonstrated by *F. occidentalis* underlines the need for judicious use of essential oils as part of broader pest control programs.

**Abstract:**

*Frankliniella occidentalis* is among the most economically significant pests of greenhouse crops, whose resistance to conventional insecticides has created demand for biopesticides such as essential oils. We assessed the fumigant toxicity of linalool against *F. occidentalis*, *F. insularis*, and *Solanum lycopersicum*. Thrips were fumigated with polyacrylamide hydrogels containing either (*R*)-linalool, (*S*)-linalool, racemic linalool, or a binary mixture of (*R*)-linalool with one of twelve adjuvants (i.e., peppermint, cedarwood, neem, clove, coconut, jojoba, soybean, olive, α-terpineol, 1,8-cineole, trans-anethole, or (*R*)-pulegone). *Solanum lycopersicum* seedlings were exposed to (*R*)-linalool or a mixture of (*R*)-linalool and peppermint oil via conditioned hydrogels or foliar spray. For *F. insularis*, (*R*)-linalool was more toxic than (*S*)-linalool, with LC_50_ values of 11.7 mg/L air and 16.7 mg/L air, respectively. Similarly for *F. occidentalis*, (*R*)-linalool was more toxic than (*S*)-linalool, with LC_50_ values of 29.0 mg/L air and 34.9 mg/L air, respectively. Peppermint oil and α-terpineol were the only synergists, while the other adjuvants exhibited varying degrees of antagonism. All seedling treatments demonstrated phytotoxicity, but symptoms were most severe for foliar sprays and mixtures containing peppermint oil. While hydrogels conditioned in linalool may be a favorable substitute to conventional insecticides, the cross-resistance demonstrated herein indicates that expectations should be metered.

## 1. Introduction

*Frankliniella occidentalis Pergande (Thysanoptera: Thripidae)*, the western flower thrips (WFT), is one of the most significant agricultural pests globally. This pest has the ability to inflict a high degree of damage on a range of crops encompassing more than 500 species spanning 50 plant families [1]. This includes ornamentals (e.g., orchid, rose, chrysanthemum, etc.) and cultivated crops (e.g., tomato, cucumber, lettuce, etc.) [2]. *Frankliniella occidentalis* are the most effective vectors of tospoviruses such as tomato spotted wilt virus (TSWV) and impatiens necrotic spot virus [3,4,5]. Tomato spotted wilt virus alone causes USD 1 billion/year in crop damage globally [6]. This estimate does not include the direct damage caused by the thrips, and it further underestimates present-day losses as *F. occidentalis* has continued to expand its geographical range to become nearly cosmopolitan [7,8]. In contrast, *Frankliniella insularis Franklin (Thysanoptera: Thripidae)* is a relatively minor pest of leguminous crops [9]. Its inability to vector tospoviruses, paired with its narrow host range, has resulted in a lower level of insecticide exposure and insecticide resistance [10].

*Frankliniella occidentalis* has elicited chemical control mainly in the form of organophosphates, carbamates, pyrethroids, and some newer insecticide chemistries. However, the efficacy of these insecticides is unsustainable and fleeting. Some synthetic insecticides can induce genotoxic, mutagenic, and carcinogenic effects in humans [11,12,13]. Due to their hydrophobic nature, these insecticides are readily absorbed into organic tissue and persist for long periods in soil and water [14]. Furthermore, 100-fold resistance can develop in as few as 20 generations in thrips [15,16].

These issues with conventional insecticides for thrips management have created a demand for alternative control methods against thrips. Linalool, an alcoholic monoterpene, is produced by members of the Lamiaceae, Rutaceae, and Lauraceae. This molecule has long been sought after for its unique odiferous and culinary properties but it has also been found to possess insecticidal properties [17]. Relatively low concentrations are needed to achieve repellency, mortality [18,19], and reduced feeding or oviposition [20]. However, its spray application can be less effective because of linalool’s volatility [21].

Insecticides are available in an array of formulations (polymer release, foliar sprays, aerosols, ultra-low volume, etc.) that vary in efficacy with the insecticide and pest in question. In agriculture, fumigation can refer to various pre- and post-harvest pest control protocols (soil fumigation, commodity fumigation, crop fumigation) [22]. The present work refers to fumigants as organic, volatile compounds that form vapors above 5 °C applied via polymer release. Incorporating essential oils into polymer, such as hydrogel matrices, allows them to retain their toxicity while slowing their volatilization. This “slow-release” allows the essential oil to persist in plantings so that it can control existing and incipient populations. Continuous exposure of the target pest attainable through pesticides’ release from polymer carriers is necessary considering that thrips’ thigmotactic behavior and resistance of eggs and pupal stages to pesticides [23] can increase the likelihood that they survive individual applications. Picard et al. 2012 [24] demonstrated that polymer matrices of alginate and methyl cellulose conditioned in essential oils repelled *F. occidentalis* for a longer period than treatment solutions lacking these polymers. Hydrogels have the added benefit of stabilizing linalool against environmental degradation (e.g., light, air, and humidity), reducing mammalian toxicity and human mucous membrane irritation, reducing phytotoxicity or fish toxicity, reducing evaporation and leaching, and reducing environmental pollution and drift [25,26]. These polymers are remarkably varied in their applications, also functioning as vehicles to deliver liquid baits to social insects [27,28].

In this study, we assessed the potential of linalool as a biopesticide against two species of thrips (*F. occidentalis* and *F. insularis*) by comparing the toxicity of linalool conditioned into polyacrylamide hydrogels of varying degrees of saturation and their efficacy with adjuvants. Additionally, *Solanum lycopersicum* Linneaus (Solanaceae) seedlings were exposed to the same oils, and applied as fumigants or foliar sprays to assess their phytotoxicity.

## 2. Materials and Methods

### 2.1. Test Organisms

*Frankliniella occidentalis* were collected from a *Cucumis sativus* Linneaus (Cucurbitceae) greenhouse in Waimea, Hawaii, as described by Nicholas and Follett (2018) [29]. Rearing chambers for *F. occidentalis* consisted of cylindrical plastic jars (12 cm diameter × 17 cm height) whose caps were fitted with wire mesh to allow ventilation. Each rearing chamber was provided with cabbage, *Brassica oleracea* Linnaeus (Brassicaceae), leaves (8 cm × 8 cm) provisioned with 0.1 mL of honey. *Frankliniella occidentalis* were deposited into the chambers with a No. 6 paintbrush and allowed to feed and oviposit for 3-day intervals. At each interval, cabbage leaves were transferred to emerging chambers, and rearing chambers were provisioned with new cabbage leaves smeared with honey. Upon eclosion, nymphs were transferred from emerging chambers to rearing chambers. The process was repeated ad infinitum. Both chambers were maintained at 26 ± 2 °C and 80 ± 5% RH under a 16:8 L:D photoperiod.

*Frankliniella insularis* were collected from a hedgerow of *Malvaviscus arboreus* dill (Malvaceae) in Honolulu, Hawaii (21.297111, −157.819151), and deposited in jars. Each rearing chamber was provisioned with snow pea pods smeared with 0.1 mL of honey. The transfer of nymphs from emerging chambers and rearing chambers followed the same protocol as *F. occidentalis*. Both chambers were maintained at 26 ± 2 °C and 80 ± 5% RH under a 16:8 L:D photoperiod.

*Solanum lycopersicum* seeds were purchased from Koolua Farmers, Honolulu, Hawaii. Empty and undeveloped seeds were discarded by floating in tap water. Seeds were sprouted in potting soil in the greenhouse at 26 ± 2 °C and 65 ± 5% RH.

### 2.2. Chemical Preparation

Polysorbate 20, both (*S*)- and (*R*)-enantiomers of linalool, 1,8-cineole, α-terpineol, trans-anethole, and (*R*)-pulegone, were purchased from Sigma–Aldrich (St. Louis, MO, USA). Clove, coconut, cedarwood, and peppermint oil were purchased from Carolina Biological Supply Company (Burlington, NC, USA). Neem oil was purchased from Monterey Lawn and Garden Products, Incorporated (Fresno, CA, USA). Jojoba, olive, and soybean oil were purchased from local grocery retailers. For hydrogel saturation tests, the dilutions of (*S*)-linalool (≥97%), (*R*)-linalool (≥95%) and polysorbate 20 included 0, 4, 8, 12, 16, 20, 24, 28, 32, 36, 40, 44, 48, and 52 mg/L air. For adjuvant tests, binary mixtures of essential oils were prepared by mixing the LC_50_ of (*R*)-linalool for each thrips species with varying concentrations (0, 6, 12, 18, 24, and 30 mg/L air) of the adjuvant. Pure distilled water (0 mg/L air essential oil) acted as negative controls for thrips assays. Dilutions were prepared in distilled water, with the volume of (*R*)-linalool + volume of the adjuvant added at a ratio of 1:1 to the volume of polysorbate 20.

For the phytotoxicity assay, three types of dilutions were assembled: pure (*R*)-linalool, (*R*)-linalool with peppermint oil, and pure distilled water (negative control). The (*R*)-linalool was prepared at concentrations below, at, and above its LC_50_ to *F. occidentalis*: 21.56 mg/L air, 29.00 mg/L air and 36.89 mg/L air, respectively. Similarly, (*R*)-linalool with peppermint oil was also prepared at three concentrations of peppermint oil, but (*R*)-linalool was maintained at 29.00 mg/L air for each: 3.98 mg/L air, 11.90 mg/L air and 19.90 mg/L air, respectively. Polysorbate 20 was added in a 1:1 ratio to the total volume of essential oil in each solution.

### 2.3. Thrips Bioassays

**Figure 1:**
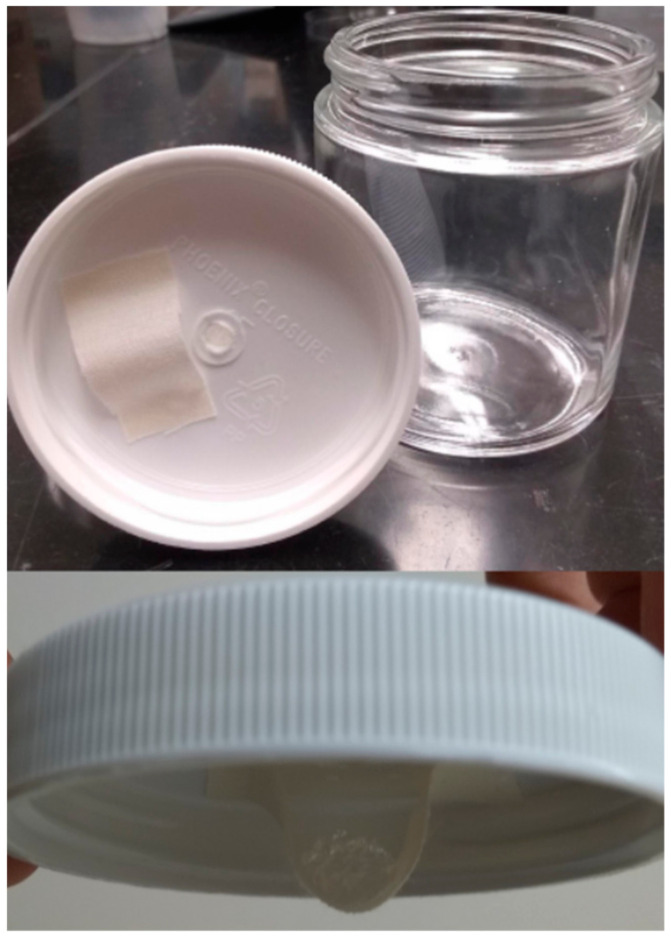
Thrips fumigation chamber with hydrogel “pouch”.

Each fumigation chamber consisted of a glass jar (5.8 cm diameter × 6.8 cm height) provisioned with either an *M. arboreus* petal (for *F. insularis*) or piece of cabbage (for *F. occidentalis*). For ventilation, 2 mm holes were melted into the caps of each jar with 4 mm squares of mesh cloth glued on top. To create a structure from which to suspend the hydrogels, the mesh cloth was cut into 5 cm × 1.5 cm rectangles. The shorter ends of these rectangles were glued to the inside of the plastic cap of each jar 4 cm apart from each other such that they formed “pouches” into which the hydrogels were inserted (Figure 1).

Polyacrylamide was purchased as Miracle-Gro^TM^ Water Storing Crystals (Miracle-Gro Lawn Products, Marysville, OH, USA). Three series of hydrogels were evaluated for both enantiomers, each for a different level of hydrogel saturation. Series one represented polyacrylamide saturated in 100-fold of its volume (0.1 mL/mg polyacrylamide), series two represented 200-fold saturation (0.2 mL/mg polyacrylamide), and series three represented 300-fold saturation (0.3 mL/mg polyacrylamide). Polyacrylamide crystals (0.01 g per Petri dish) were weighed using Ohaus PR224 PR Series Analytical Balance (Ohaus Corporation, Parsippany, NJ, USA), and the corresponding linalool dilution (from Chemical Preparation) was pipetted into each Petri dish. The Petri dishes were capped, and the hydrogel crystals were conditioned to allow uptake of the volatile compound by the hydrogel for at least 24 h prior to bioassays.

For each fumigation treatment, 0.3 g of conditioned hydrogel was inserted into the “pouch”. Ten adult thrips were carefully transferred with a No. 6 paintbrush into each chamber. Each treatment was performed in replicates of four. Mortality was assessed after 24 h by counting the number of live and dead thrips. Individuals were determined to be dead if they did not respond to mechanical stimuli or exhibited *rigor mortis*.

### 2.4. Phytotoxicity Assays

For each hydrogel treatment, 2.70 g of polyacrylamide was placed in each plastic jar (7.6 cm diameter × 10 cm height). The corresponding dilutions (from Chemical Preparation) were poured into jars and mixed for 5 min. The caps were screwed on, and the hydrogels with essential oil mixtures were conditioned for at least 24 h prior to the bioassays.

Upon emergence of the epicotyl, seedlings were randomly divided into groups of 10 and placed into black plastic trays with plastic humidity domes (63 cm length × 33 cm width × 22 cm height) fitted overhead. Plants were watered before fumigation, as previous experience has shown that plants fumigated under dry conditions are liable to be injured [30]. For hydrogel treatments, a plastic dish containing 64 g of conditioned hydrogel was placed in the center of the tray. For the foliar spray treatments, seedlings were sprayed with treatment solutions until evenly wetted. Phytotoxicity was assessed after 1 week of exposure by measuring the length of root and hypocotyl. Lengths of 0 cm were recorded for seedlings killed by treatments.

### 2.5. Nuclear Magnetic Resonance (NMR) Sample Preparation and Analysis

NMR analysis was performed to determine if the dehydration products of linalool (i.e., ocimene and myrcene) were present in the treatment solutions. Five samples were prepared for NMR analysis: 11.7 mg/L (*R*)-linalool and 24.0 mg/L neem, 11.7 mg/L (*R*)-linalool and 30.0 mg/L neem, 11.7 mg/L (*R*)-linalool and 18.0 mg/L cedarwood oil, 11.7 mg/L (*R*)-linalool and 24.0 mg/L cedarwood oil, and 11.7 mg/L (*R*)-linalool and 18.0 mg/L (*S*)-linalool.

Each sample was prepared directly in the corresponding NMR tube, consisting of 600 µL D_2_O and polysorbate 20 added in a 1:1 ratio to the total volume of essential oil. Deuterated chloroform was added to improve the immiscibility of the oils. The 1H NMR experiments were carried out on Agilent 400 DD2 and 600 spectrometers (Agilent Technologies, Palo Alto, CA, USA). Each experiment was performed at room temperature with 256 scans per sample.

### 2.6. Statistical Analysis

Data were analyzed using SPSS version 26.0 (IBM Corp., Armonk, NY, USA) [31]. Average mortality data for each treatment from thrips bioassays (hydrogel saturation tests and adjuvant tests) were subjected to probit analysis [32] to obtain the lethal concentration (LC) values. LC values were considered significantly different when their 95% confidence intervals did not overlap.

The binary interactions of essential oils against adult *F. insularis* and *F. occidentalis* were quantified according to the synergism ratio (SR) as described by Chadwick 1961 [33] and Metcalf 1967 [34]:

SR = LC_50_ of pure (*R*)-linalool/LC_50_ of mixture (1)

A synergism ratio of 1.0 indicated that the toxicity of pure (*R*)-linalool was equal to the toxicity of the mixture of (*R*)-linalool and the adjuvant. A value >1.0 indicated synergism, a value <0.5 was classified as strong antagonism, and a synergism ratio >0.5 but <1.0 was classified as weak antagonism.

Root and hypocotyl lengths were subjected to one-way analysis of variance (ANOVA) to identify significant differences due to type of essential oil (pure (R)-linalool or (R)-linalool with peppermint oil). Mean values were then separated with Tukey’s honestly significant difference (HSD) test at the 0.05 level of significance.

## 3. Results

### 3.1. Hydrogel Saturation Tests

For *F. insularis*, the median lethal concentrations (LC_50_) for hydrogels conditioned in (R)-linalool with 100-, 200-, and 300-fold saturation were 11.7 (10.0–13.4), 18.1 (14.7–21.4), and 20.6 (19.3–21.8) mg/L air, respectively, while the LC_90_ values were 18.0 (16.0–21.2), 30.2 (26.2–37.4), and 30.2 (28.4–32.4) mg/L air, respectively (Table 1).

For *F. occidentalis*, the LC_50_ values for hydrogels conditioned in (*R*)-linalool with 100-, 200-, and 300-fold saturation were 29.0 (27.1–30.9), 35.1 (33.7–36.5), and 38.9 (37.4–40.4) mg/L air, respectively, while the LC_90_ values were 36.5 (34.1–40.3), 47.7 (45.6–50.3), and 52.8 (50.4–55.7) mg/L air, respectively (Table 1).

No mortality occurred in untreated controls (hydrogels conditioned in pure distilled water). For almost all treatments, (*R*)-linalool conditioned in the least saturated (100-fold) hydrogels of either enantiomer was the most toxic, and the *R* enantiomer was more toxic than the *S* enantiomer.

### 3.2. Adjuvant Tests

For *F. insularis*, peppermint oil was the only synergist, with an LC_50_ of 10.7 (7.3–12.8) and LC_90_ of 16.2 (14.4–18.2) mg/L air (Table 2). α-Terpineol induced 100% mortality at all tested concentrations. Several adjuvants (i.e., (*S*)-linalool, cedarwood, neem, and 1,8-cineole) behaved as mild antagonists. The remaining adjuvants (i.e., trans-anethole, (*R*)-pulegone, clove, coconut, jojoba, olive, and soybean) behaved as severe antagonists (Table 3).

Similar trends were observed in *F. occidentalis* relative to pure (*R*)-linalool with LC_50_ of 29.0 mg/L air. α-terpineol was the only synergist, with an SR ratio of 1.090 (Table 3). While peppermint oil was not classified as a synergist in *F. occidentalis*, it had the second greatest insecticidal efficacy with an LC_50_ of 30.0 (24.5–33.0) and an LC_90_ of 43.4 (41.0–47.4) mg/L air (Table 2). Another four adjuvants (i.e., (*S*)-linalool, 1,8-cineole, trans-anethole, and (*R*)-pulegone), behaved as mild antagonists while the remaining adjuvants (i.e., cedarwood, neem, clove, coconut, jojoba, olive, and soybean) behaved as severe antagonists (Table 3). No mortality was found in untreated control (hydrogels conditioned in pure distilled water).

### 3.3. Phytotoxicity Assays

The observed symptoms of phytotoxicity included reduced root and hypocotyl lengths, dieback, stunting, depigmentation, and death. The most severe phytotoxicity was recorded for the peppermint oil mixture. Significant differences were found on the root and hypocotyl lengths of seedlings sprayed with all concentrations of (*R*)-linalool with peppermint oil and the highest concentrations of (*R*)-linalool, as compared with that of control (*p* < 0.05). For hydrogel fumigation, no significant difference was found on the hypococtyl lengths of seedlings after treating with all concentrations of (*R*)-linalool whereas significantly shorter hypococtyls were recorded at the two highest concentrations of (*R*)-linalool with peppermint oil, as compared with that of control (*p* < 0.05).

### 3.4. NMR Analysis

**Figure 2:**
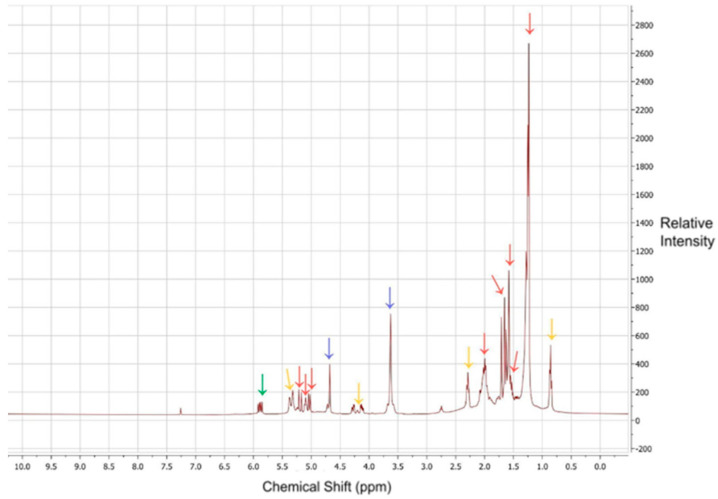
NMR spectra for sample 1. The green arrow indicates peaks for potential dehydration products, red arrows indicate peaks for (*R*)-linalool, blue arrows indicate peaks for polysorbate 20 and yellow arrows indicate peaks for triglycerides and similarly unsaturated fatty acids.

Dehydration products (i.e., ocimene and myrcene) were not detected in samples three to five, but they were detected in samples one and two as peaks from 5.80 to 5.95 ppm. These two samples had nearly identical spectra; therefore, only sample one (Figure 2) is shown because it had peaks of greater intensity in this range. The peaks for ocimene and myrcene had low intensity, indicating that the dehydration products did not constitute a significant portion of the samples (<1%). Linalool formed strong peaks throughout the spectra at 1.27, 1.56, 1.60, 1.68, 1.85, 5.02, 5.11, and 5.20 ppm. Polysorbate 20 produced a strong peak at 3.64 ppm.

## 4. Discussion

The enantioselective toxicity of linalool toward thrips was demonstrated in this study: (*R*)-linalool was 35% more toxic than (*S*)-linalool to *F. insularis* and 18.5% more toxic to *F. occidentalis*. Enantioselective toxicity to animals and plants is well documented in many other stereoisomeric compounds [35,36,37,38,39]. While linalool can inhibit multiple targets in the insect nervous system (acetylcholinesterase, gamma-aminobutyric acid, etc.), acetylcholinesterase (AChE) appears to be the primary inhibitory target [40,41]. This enantioselective toxicity is thought to be the result of (*R*)-linalool being a more potent AChE inhibitor than (*S*)-linalool. Linalool’s ability to bind to AChE is dependent on the alcoholic monoterpenoid’s ability to form intermolecular forces (i.e., hydrogen bonds, hydrophobic interactions, and Van der Waals) with certain amino acids in AChE [42,43,44]. It is possible that enantioselective intermolecular forces occur in the (*R*)-linalool-AChE complex but not in the (*S*)-linalool-AChE complex. Since the orientation of the bond of the hydroxyl group to the 3′ carbon [45] was the only difference between these enantiomers, it was likely responsible for the enantioselective formation of intermolecular forces and the consequent steric hindrance (or lack thereof) for the inhibitor-enzyme complexes.

Synergisms resulted when both components of a binary mixture had unique inhibitory targets. The (*R*)-linalool and peppermint oil synergism serves as an example, with a synergistic ratio >1.0 (Table 3). Linalool inhibits AChE, while peppermint oil inhibits gamma-aminobutyric acid (GABA) [46,47]. When mixed together and applied to *F. insularis*, the mortality was higher than that of (*R*)-linalool applied individually because the simultaneous inhibition of AChE and GABA was more damaging than only AChE inhibition [48]. Alternatively, these synergisms may be a reflection of the exceptional insecticidal activity of the adjuvants, rather than their interaction with (R)-linalool.

Weak antagonistic mixtures were those with synergistic ratios <1.0 but >0.5. This phenomenon is thought to occur due to overlapping modes of action of (*R*)-linalool and the adjuvant, with the racemate serving as an example in both species (i.e., LC_50_ of 16.5 mg/L air for *F. insularis* and LC_50_ of 45.8 mg/L air for *F. occidentalis*). The enantiomers of linalool were expected to have similar modes of action, considering their structural similarity [45]. This overlap in inhibitory targets did not increase mortality because only AChE function was diminished. It was possible that the components of this binary mixture shared not only the same inhibitory target but also the same active site on that target.

Another possible explanation for the mild antagonisms described above was the decomposition of linalool under low pH, leading to a reduction in its insecticidal efficacy [49]. Linalool is prone to dehydration because it is not only a tertiary alcohol, but also an allylic alcohol. Tertiary alcohols are more reactive because the presence of additional alkyl groups increases the inductive effect. The charge density around the tertiary carbon increases, and consequently in the C-O bond, which facilitates the cleavage of that bond [50]. Allylic carbons are more reactive than simple alkanes due to their proximity to adjacent π systems [51]. Theoretically, solutions containing neem, cedarwood or *(S*)-linalool as adjuvants cause (*R*)-linalool to dehydrate into a mixture of products (i.e., myrcene and ocimene) via unimolecular elimination [52]. As the proportion of (*R*)-linalool decreases while ocimene and myrcene increases, the insecticidal activity is reduced because these dehydration products have relatively less insecticidal activity [53,54].

However, the absence of strong peaks for dehydration products in the NMR spectra of these solutions (Figure 2) revealed that ocimene and myrcene did not occur in samples containing cedarwood oil or (*S*)-linalool. While dehydration products were detected in solutions containing neem oil as an adjuvant, these products did not occur as a significant enough proportion (<1%) to be responsible for the observed reduction in insecticidal efficacy. Nonetheless, their presence highlights the value of detecting biopesticide decomposition. Other adjuvants may influence insecticidal activity by creating conditions conducive to linalool’s decomposition. Therefore, identification of the characteristics of these adjuvants can help to exclude potential antagonists.

In contrast, strong antagonisms arose from the behavior of five oils (i.e., clove, coconut, jojoba, soybean, and olive oil) as fixatives. These oils produced synergistic ratios <0.5 (Table 3), high boiling points and low vapor pressures. Additionally known as carrier oils, they depress the volatility of other components in the mixture [55,56]. However, the combined volatility depression of the hydrogel matrix and the fixative rendered (*R*)-linalool so nonvolatile that mortality was significantly reduced for two oils (i.e., olive and clove oil) and completely reduced for three oils (i.e., coconut, jojoba, and soybean oil). Van der Waals and dipole–dipole interactions between (*R*)-linalool and the fixative potentially contributed to this non-volatility [57]. Some oils (i.e., cedarwood and neem) behaved as weak antagonists for *F. insularis*, but were strong antagonists for *F. occidentalis*, and also vice versa (i.e., trans-anethole and (*R*)-pulegone). The former may be a result of the heightened resistance of *F. occidentalis* to a range of pesticide compounds. The latter phenomenon requires in vitro studies of insect nervous systems to clarify the mechanism responsible for this discrepancy.

The LC_50_ of WFT to both enantiomers of linalool and the adjuvant mixtures was consistently higher than that of *F. insularis*. For example, pure (*R*)-linalool had an LC_50_ of 11.7 mg/L air in *F. insularis* and an LC_50_ of 29.0 mg/L air in *F. occidentalis* (Table 2).

Considering that essential oil fumigation is a relatively novel method, it is improbable that WFT acquired resistance through previous exposure. However, there are records of WFT resistance to synthetic pesticides, many of which share a mode of action with the essential oils in this study [8,58,59,60,61,62,63]. Resistance has been documented in closely related species in Hawai’i [64,65] and in WFT populations in California and Japan [8,58,59]. While WFT resistance may have arisen on the archipelago independently of immigration, it is possible that the trans-Pacific trade of plant material spread WFT populations bearing resistance genes to Hawai’i [66].

Of the 19 synthetic pesticides registered for the management of thrips in the United States, at least five (i.e., abamectin, acephate, chlorpyrifos, methiocarb, and spinosad) have one or both of the same modes of action as the essential oils tested in our study [66]. There are multiple mechanisms potentially responsible for the increased resistance of WFT compared with *F. insularis*. However, the majority of insecticide-resistant WFT cases result from metabolic detoxification (e.g., cytochrome P450s) [67]. Resistance mechanisms can co-occur and synergize. For example, thigmotaxis may result in a reduced rate of entry of linalool into thrips bodies, enabling metabolic detoxification to occur without P450s being overwhelmed [60]. While exposure to insecticides undoubtedly plays a role in the development of resistance in WFT, the highly polyphagous nature of this species might also be responsible for its predisposition to metabolic detoxification [60]. Due to its widely varied diet, WFT often need to detoxify allelochemicals produced by some of their host plants. *Frankliniella insularis* has a narrower host range; therefore, there is reduced selective pressure for metabolic detoxification.

*Orius strigicollis* Poppius (Hemiptera: Anthocoridae) is one the primary biocontrol agents of thrips. Kim et al. (2014) and Yi et al. (2006) both found this natural enemy to possess a greater degree of resistance to essential oil fumigation than *Thrips palmi* Karny (Thysanoptera: Thripidae) [68,69]. *Neoseiulus californicus* McGregor (Acari: Phytoseiidae) is another natural enemy of thrips shown to have a degree of resistance to essential oil fumigation [70,71]. However, whether the resistance of these biocontrol agents is greater than that demonstrated by *F. occidentalis* is yet to be investigated.

The lack of significant differences in the root and hypocotyl lengths of seedlings exposed to (*R*)-linalool and peppermint oil as fumigants indicates that both types of oils induced the same level of phytotoxicity via fumigation. However, peppermint oil induced greater phytotoxicity than pure (*R*)-linalool when applied as foliar sprays (Table 4). Considering that tomatoes are relatively vulnerable to phytotoxicity following direct exposure to essential oils [72,73,74,75], the decreased hypocotyl and root lengths of seedlings treated with the peppermint oil spray were expected. Essential oil mixtures applied as fumigants behave similar to gases, filling the volume of their container. This behavior allows the essential oil to make contact with thrips while minimizing its direct contact with the plant. In contrast, essential oils applied as sprays are more concentrated on the plant tissue, resulting in more severe phytotoxicity. However, the mechanism underlying the increased phytotoxicity of the peppermint oil mixture compared with pure (*R*)-linalool is less clear.

Essential oils can induce phytotoxicity through disruptions in osmoregulation, membrane potential, mitochondrial respiration, phytohormones, microtubules, genotoxicity and the generation of reactive oxygen species [76]. Peppermint oil has been implicated in more of these mechanisms than linalool, which may be responsible for its increased phototoxicity [77,78,79].

## 5. Conclusions

We conducted a series of fumigation assays to assess the vulnerability of *F. occidentalis* and *F. insularis* to fumigation with essential oils via polymer release. In phytotoxicity assays, *S. lycopersicum* seedlings were screened for their sensitivity to the most potent fumigants, as determined from thrips bioassays. Hydrogels conditioned in linalool at the lowest saturation were the most effective, which had the added benefit of requiring the least volume of essential oil. Both species of thrips demonstrated enantioselective toxicity to linalool. However, *F. occidentalis* was significantly more resistant to these treatments than *F. insularis*. Fumigation of *S. lycopersicum* with the same concentrations of oils required to control thrips caused phytotoxicity. Phytotoxicity was more severe in seedlings exposed to these oils as foliar sprays, affirming the utility of polymer release. These findings underline the need to assess the potential of synthetic synergists (e.g., piperonyl butoxide, diethyl maleate, etc.) applied in tandem with essential oils to disarm insecticide resistance in *F. occidentalis*. Furthermore, the vulnerability of natural enemies to essential oils should be evaluated to determine if they surpass the resistance of *F. occidentalis* demonstrated herein.

## Figures and Tables

**Table 1:** LC_50_ and LC_90_ values for different treatments of *F. insularis* and *F. occidentalis* (*n* = 4).

Treatment	*F. insularis*						*F. occidentalis*					
	LC_50_ in mg/L Air (LCL–UCL)	LC_90_ in mg/L Air (LCL–UCL)	Slope ± SE	df	χ^2^	*p* Value	LC_50_ in mg/L Air (LCL–UCL)	LC_90_ in mg/L Air (LCL–UCL)	Slope ± SE	df	χ^2^	*p* Value
100× (*R*)-linalool	11.7 (10.0–13.4)	18.0 (16.0–21.2)	0.20 ± 0.04	6	10.02	0.12	29.0 (27.1–30.9)	36.5 (34.1–40.3)	0.17 ± 0.03	11	24.20	0.01
200× (*R*)-linalool	18.1 (14.7–21.4)	30.2 (26.2–37.4)	0.11 ± 0.02	9	33.40	<0.001	35.1 (33.7–36.5)	47.7 (45.6–50.3)	0.10 ± 0.01	14	9.73	0.78
300× (*R*)-linalool	20.6 (19.3–21.8)	30.2 (28.4–32.4)	0.13 ± 0.02	9	8.95	0.44	38.9 (37.4–40.4)	52.8 (50.4–55.7)	0.09 ± 0.01	14	6.55	0.95
100× (*S*)-linalool	16.7 (16.0–17.3)	19.3 (18.5–20.6)	0.47 ± 0.10	6	0.43	0.10	34.9 (33.5–36.3)	46.9 (44.9–49.3)	0.12 ± 0.02	14	6.39	0.96
200× (*S*)-linalool	17.2 (16.6–17.9)	19.7 (18.9–21.0)	0.52 ± 0.15	6	0.20	0.10	43.2 (41.6–44.9)	58.9 (56.4–62.0)	0.08 ± 0.01	16	10.41	0.84
300× (*S*)-linalool	32.3 (31.0–33.6)	42.4 (40.5–45.0)	0.13 ± 0.02	11	10.60	0.48	42.1 (40.8–43.5)	53.5 (51.6–55.9)	0.11 ± 0.02	16	11.20	0.80

LC_50_ = concentration to kill 50% of thrips, LC_90_ = concentration to kill 90% of thrips, LCL–UCL = lower confidence limit to upper confidence limit, SE = standard error, df = degrees of freedom, χ^2^ = Pearson goodness-of-fit test. *p* values derived from chi-squared test. There are varied numbers of concentrations tested for each adjuvant.

**Table 2:** LC_50_ and LC_90_ values for different adjuvants mixed with (*R*)-linalool of *F. insularis* and *F. occidentalis* (*n* = 4).

Adjuvant	*F. insularis*						*F. occidentalis*					
	LC_50_ in mg/L Air (LCL–UCL)	LC_90_ in mg/L Air (LCL–UCL)	Slope ± SE	df	χ^2^	*p* Value	LC_50_ in mg/L Air (LCL-UCL)	LC_90_ in mg/L Air (LCL-UCL)	Slope ± SE	df	χ^2^	*p* Value
Pure (*R*)-linalool	11.7 (10.0–13.4)	18.0 (16.0–21.2)	0.20 ± 0.04	6	10.02	0.12	29.0 (27.1–30.9)	36.5 (34.1–40.3)	0.17 ± 0.03	11	24.20	0.01
Peppermint with (*R*)-linalool	10.7 (7.3–12.8)	16.2 (14.4–18.2)	0.23 ± 0.08	4	3.36	0.50	30.0 (24.5–33.0)	43.4 (41.0–47.4)	0.10 ± 0.04	4	3.29	0.51
(*S*)-linalool with (*R*)-linalool	16.5 (11.9–18.4)	21.5 (19.4–28.5)	0.26 ± 0.09	4	7.28	0.12	45.8 (44.3–47.3)	58.2 (56.1–61.0)	0.10 ± 0.02	9	7.97	0.54
Cedarwood with (*R*)-linalool	17.7 (15.7–19.3)	26.4 (24.6–29.0)	0.15 ± 0.04	4	3.11	0.54	63.8 (60.3–67.5)	80.5 (75.4–89.0)	0.08 ± 0.01	12	36.72	<0.001
Neem with (*R*)-linalool	20.7 (13.8–25.3)	37.7 (32.4–48.1)	0.08 ± 0.02	5	10.53	0.06	73.8 (70.8–76.7)	105.4 (101.1–110.9)	0.04 ± 0.01	15	15.81	0.40
Clove with (*R*)-linalool	23.6 (19.4–27.0)	30.2 (26.9–40.3)	0.19 ± 0.05	4	13.35	0.01	63.3 (61.3–65.3)	82.4 (79.3–86.2)	0.07 ± 0.01	12	7.80	0.80
1,8-Cineole with (*R*)-linalool	20.8 (15.3–24.6)	35.9 (31.1–46.0)	0.09 ± 0.02	6	15.44	0.01	38.8 (35.6–41.3)	58.6 (55.6–62.6)	0.06 ± 0.01	9	10.81	0.30
*trans*-Anethole with (*R*)-linalool	33.2 (31.3–35.1)	40.8 (38.1–44.3)	0.18 ± 0.03	7	11.84	0.11	53.2 (51.9–54.5)	61.9 (60.2–64.2)	0.15 ± 0.03	9	7.68	0.60
(*R*)-Pulegone with (*R*)-linalool	25.3 (21.7–28.4)	35.7 (31.9–43.4)	0.12 ± 0.02	7	22.44	<0.01	47.0 (42.9–50.5)	70.3 (65.1–78.7)	0.06 ± 0.01	10	16.96	0.08

LC_50_ = concentration to kill 50% of thrips, LC_90_ = concentration to kill 90% of thrips, LCL–UCL = lower confidence limit to upper confidence limit, SE = standard error, df = degrees of freedom, χ^2^ = Pearson goodness-of-fit test. *p* values derived from chi-squared test. There are varied numbers of concentrations tested for each adjuvant. LC_50_ values that could not be calculated due to insufficient or excessive mortality were not included.

**Table 3:** Synergistic ratios (SRs) of adjuvants to (*R*)-linalool for *F. insularis* and *F. occidentalis*.

Adjuvant	*F. insularis*	*F. occidentalis*
Peppermint and (*R*)-linalool	1.093	0.967
(*S*)-linalool and (*R*)-linalool	0.709	0.633
Cedarwood and (*R*)-linalool	0.661	0.455
Neem and (*R*)-linalool	0.565	0.393
Clove and (*R*)-linalool	0.496	0.458
1,8-Cineole and (*R*)-linalool	0.563	0.747
trans-Anethole and (*R*)-linalool	0.352	0.545
(*R*)-Pulegone and (*R*)-linalool	0.462	0.617
α-Terpineol and (*R*)-linalool	N/A	1.090

N/A = not assessed due to 100% mortality at all tested concentrations. SRs that could not be calculated due to insufficient or excessive mortality were not included.

**Table 4:** Phytotoxicity of (*R*)-linalool and peppermint oil applied as foliar spray or hydrogel fumigation to *S. lycopersicum* (*n* = 10).

Concentration (mg/L air)		Length (cm) of Seedlings (Mean ± SE)			
		Foliar Spray		Hydrogel Fumigation	
		Hypocotyl	Root	Hypocotyl	Root
Control		5.40 ± 0.14 ^a^	7.45 ± 1.23 ^a^	6.60 ± 0.19 ^a^	11.10 ± 0.91 ^a^
(*R*)-Linalool	21.56	4.55 ± 0.75 ^a^	7.10 ± 1.65 ^a^	5.90 ± 0.21 ^ab^	10.70 ± 0.59 ^ab^
	29.00	3.80 ± 0.80 ^a^	6.95 ± 0.52 ^a^	5.75 ± 0.28 ^ab^	7.95 ± 0.67 ^b^
	36.89	0.00 ± 0.00 ^b^	0.00 ± 0.00 ^b^	5.65 ± 0.30 ^ab^	7.85 ± 0.52 ^b^
(*R*)-linalool with peppermint oil	3.98	0.00 ± 0.00 ^b^	0.00 ± 0.00 ^b^	5.75 ± 0.21 ^ab^	10.95 ± 0.96 ^ab^
	11.90	0.00 ± 0.00 ^b^	0.00 ± 0.00 ^b^	5.25 ± 0.23 ^b^	9.55 ± 0.85 ^ab^
	19.90	0.00 ± 0.00 ^b^	0.00 ± 0.00 ^b^	5.45 ± 0.31 ^b^	9.30 ± 0.41 ^ab^

SE = standard error. Means followed by different letters in the same column indicate statistically significant differences (one-way ANOVA followed by Tukey’s HSD test; *p* < 0.05).

## Data Availability

The data presented in this study are available on request from the corresponding author.
